# Influence of co-existing atrial fibrillation on the efficacy of atorvastatin treatment in patients with dilated cardiomyopathy: a pilot study

**DOI:** 10.1186/1476-511X-9-21

**Published:** 2010-02-23

**Authors:** Agata Bielecka-Dąbrowa, Jan Henryk Goch, Jacek Rysz, Marek Maciejewski, Ravi Desai, Wilbert S Aronow, Maciej Banach

**Affiliations:** 1 Department of Hypertension, Chair of Nephrology and Hypertension, Medical University of Lodz, Poland; 2 Department of Nephrology, Hypertension and Family Medicine, Chair of Nephrology and Hypertension, Medical University of Lodz, Poland; 3 Department of Cardiology, 1st Chair of Cardiology and Cardiac Surgery, Medical University of Lodz, Poland; 4 Division of Gerontology, Geriatrics and Palliative Care, University of Alabama at Birmingham, Birmingham, AL, USA; 5 Cardiology Division, New York Medical College, Valhalla, NY, USA

## Abstract

**Introduction:**

The aim of the study was to assess the influence of co-existing atrial fibrillation (AF) on inflammatory condition factors, left ventricular function, clinical course and the efficacy of statin treatment of congestive heart failure in the course of dilated cardiomyopathy (DCM).

**Material and methods:**

In a prospective, randomized, open-label study, 69 patients with DCM and left ventricular ejection fraction (LVEF) ≤40% were divided into two groups, with and without AF, who were treated according to the recommended standards. 68% of patients from the group with AF and 59% of patients from the group without AF were administered atorvastatin 40 mg daily for 8 weeks and 10 mg for next 4 months. Clinical examination with the assessment of body mass index (BMI) and waist size were followed by routine laboratory tests, measurement of concentration of tumor necrosis factor (TNF-α), interleukin-6 (IL-6), and IL-10 in blood plasma, N-terminal pro-brain natriuretic peptide (NT-proBNP) concentration in blood serum, echocardiographic examination, and the assessment of exercise capacity in 6-minute walk test (6-MWT). After six months, morbidity rate and the number of heart failure hospitalizations were also observed.

**Results:**

In the whole population of patients, a significantly higher concentration of NT-proBNP was observed in the AF group (2669 ± 2192 vs 1540 ± 1067, p = 0.02). After statin treatment, in patients with DCM and co-existing AF, higher values of NT-proBNP and IL-6 were observed compared to non-AF patients (1530 ± 1054 vs 1006 ± 1195, p = 0.04 and (14.16 ± 13.40 vs 6.74 ± 5.45, p = 0.02, respectively).

**Conclusion:**

In patients with DCM and co-existing AF, a weaker effect of atorvastatin concerning the reduction of IL-6 and NT-proBNP concentration was observed than in patients without atrial fibrillation.

**Trials Registration:**

(*ClinialTrial.gov *No.: NCT01015144)

## Introduction

Atrial fibrillation (AF) is the most commonly encountered cardiac arrhythmia and is directly or indirectly responsible for considerable mortality, morbidity and health care burden [1,2]. Hypertension, coronary artery disease (CAD), and cardiomyopathy represent the most prevalent underlying pathologies of AF and congestive heart failure (HF), implying a coincidence of both in many patients [2-4]. According to the European Society of Cardiology (ESC) 2007 guidelines [5], dilated cardiomyopathy (DCM) is recognized based on dilation and systolic dysfunction of the left ventricle unless a patient simultaneously suffers from CAD, hypertension, valvular heart disease or congenital heart disease which is so significant that it leads to an observed pathology of the myocardium. Within the last few years, more and more evidence has been presented that autoimmunological processes, cellular as well as humoral ones, are involved in the pathogenesis of DCM [6].

The prevalence of AF with a progressive degree of congestive HF is increasing, as judged by New York Heart Association (NYHA) functional class [7,8]. Moreover, the presence of congestive HF has been identified as one of the most powerful independent predictors of AF, with a sixfold increase in relative risk of its development [7,8]. On the other hand, AF can cause or significantly aggravate symptoms of congestive HF in previously asymptomatic or well-compensated patients [3,7]. Recent investigations of the physiological and structural changes of the atrial myocardium ("electrical and structural remodelling") have shown that neurohumoral activation, fibrosis, and apoptosis are demonstrable with both diseases. On the other hand, experimental data suggest that the substrates of AF in congestive HF are different from those of pure atrial tachycardia-related forms of AF [3,7,8].

Statins inhibit the enzyme of 3-hydroxy-3-methylglutaryl coenzyme A (HMG-CoA) reductase and, at the same time, the synthesis of cholesterol [9]. The pleiotropic effects of statins may be connected with their basic mechanism, that is the inhibition of HMG-CoA reductase. In this mechanism, not only is the synthesis of cholesterol reduced, but production of the derivatives of mevalonic acids, isoprenoids is reduced as well [9-11]. Limiting the production of isoprenoids, statins block the function of cytoplasmic regulatory proteins, namely GTPases from the Rho protein family, such as Ras, Rac1 and Rap. As a result, they positively influence the course of intracellular reactions connected, among other things, with inhibiting the hypertrophy and remodelling of the myocardium. The blockade of Rac decreases the vascular and myocardial oxidative stress through the inhibition of nicotinamide adenine dinucleotide phosphate (NAD(P)H) oxidase activation [9-12]. The deteriorating circulatory insufficiency is characterized by increased amounts of free radicals, which may inactivate nitric oxide (NO). Therefore, additional advantages of Rho protein inhibition are also connected with the increased endothelial synthesis of nitrogen oxide and reduced expression of endothelin-1 (ET-1), which has a positive effect on endothelium function [13-15]. In addition, they inhibit the synthesis of inflammatory cytokines and chemokines, improve autonomic function, and reverse myocardial remodelling [16,17].

Because of the pleiotropic effect of statins, there are attempts to use this group of medicines also in the treatment of patients with AF accompanying DCM. Therefore, the aim of our study was to assess the influence of co-existing AF on inflammatory condition factors, left ventricular function, clinical course and the efficacy of statin treatment of cardiac insufficiency in the course of DCM.

## Materials and methods

### Study population

In a prospective, randomized study 69 patients (men and women aged 18 years or older) with DCM (according to ESC 2007 [5]) with a left ventricular ejection fraction (LVEF) ≤40% as documented by echocardiography were included. Patients had been on stable doses of HF medications for three weeks before enrollment. Mean disease duration was 2.56 ± 1.98 years. None of the patients had significant CAD (defined as >30% obstruction diagnosed during cardiac catheterization performed during the one year before enrolment) or hypertension.

The exclusion criteria were as follows: blood pressure (BP) ≥140/90 or <90/60; congenital heart disease; acquired valvular disease with the exception of mitral incompetence secondary to left ventricular dilatation; HF with NYHA class IV; statin treatment before the inclusion; preserved hyperactivity of aminotransferases with unexplained aetiology; muscle disorders which might cause drug-induced myopathy; uncontrolled diabetes; liver diseases; creatinine level >2 mg/dl and/or glomerular filtration rate (eGFR) <30 ml/min; alcohol or drug abuse; chronic inflammatory diseases; pregnancy or lactation; severe hypothyroidism; immunosuppressive treatment; operation or severe injury during the last month; vaccination during the last 3 months; heart stimulation device or implantable cardioverter defibrillator (ICD) and patients who did not provide written informed consent.

After inclusion all patients were randomized to groups A and B. It was an open-label study. Group A consisted of 41 patients (93% males) of mean age 56 ± 10 years, who were administered atorvastatin 40 mg daily for 2 months (8 weeks) and next 10 mg for 4 months. Group B consisted of 27 patients (74% males) of mean age 59 ± 14 years, in whom DCM was treated according to present standards [1] without statin therapy. Next, they were divided to 2 groups: the group with AF, and the group without AF (non-AF group). AF group consisted of 25 patients (male - n = 22, 88%) of mean age 57 ± 10 years. Non-AF group consisted of 44 patients (male - n = 37, 84%) of mean age 59 ± 13 years in whom DCM was treated according to current standards [5]. 68% (n = 17) of patients from AF group and 61% (n = 27) of patients from non-AF group were administered atorvastatin 40 mg daily for 2 months (8 weeks) and next 10 mg for 4 months.

A full clinical examination including the assessment of body mass index (BMI) and waist size was followed by routine laboratory tests, measurement of tumour necrosis factor alpha (TNF-α), interleukin 6 (IL-6), and IL-10 concentration in blood plasma, measurement of N-terminal pro-brain natriuretic peptide (NT-proBNP) concentration in blood serum, echocardiographic examination and the assessment of exercise capacity in 6-minute walk test (6-MWT). During 6-months' observation, the frequency of hospitalizations due to cardiovascular symptoms, mortality and causes of death were assessed; all patients were also classified according to NYHA class.

The consent of the Bioethics Commission of the Medical University of Lodz, Poland, number RNN/54/07/KE was obtained. Written informed consent was obtained from all patients. The research was financed from the grant of the Medical University of Lodz, Poland, No. 502-11-585.

### Biochemical tests

Blood glucose was measured with a glucose dehydrogenase method after precipitation of proteins by trichloroacetic acid. Low-density lipoprotein (LDL) and high-density lipoprotein (HDL) fractions were separated from fresh serum by combined ultracentrifugation and precipitation. Lipoprotein fraction cholesterol and triglycerides were measured enzymatically. The concentration of NT-proBNP was determined using an Elecsys 2010 analyser (Roche Diagnostics, Warsaw, Poland). After the blood was taken, the material was centrifuged; the obtained serum was frozen at the temperature of -70°C and stored in this condition until the moment of examination. The determination of NT-proBNP in blood serum was performed with the electroluminescence method with two polyclonal antibodies directed against NT-proBNP within epitope 1 (1-21 amino acids sequence) and epitope 2 (39-50 amino acids). Concentration values are given in pg/ml. Determination of IL-6 and 10 as well as TNF-α was performed with reagents from Beckman Coulter (Paris, France), using a sandwich ELISA assay (enzyme-linked immunosorbent assay) [18]. According to the recommendations of the producer, blood was taken on EDTA as an anticoagulant. After that, it was centrifuged for 15 minutes. The obtained plasma was frozen at the temperature of -70°C until the moment of examination.

### 6-minute walk test

In all patients, a 6-MWT was performed. The examination was conducted according to the following protocol:

• time of test was between 10 a.m. and 4 p.m. after usual medication;

• a 35 m flat, obstacle-free corridor, with chairs placed at either end, was used;

• patients were instructed to walk as far as possible, turning 180° every 35 m in the allotted time of 6 min;

• patients were able to rest, if necessary;

• time remaining was called every second minute;

• on completion of 6 min, patients were instructed to stop and total distance covered was calculated to the nearest metre.

### Echocardiographic assessment

Echocardiography was performed using Sonos 5500 (Agilent Technologies Inc., Hewlett Packard, Andover, USA) with a 3-11 MHz probe. Left ventricular systolic function and cardiac dimensions indexed to body surface area were determined. The heart was imaged in parasternal short axis view to obtain LV wall thickness and parasternal long axis view to measure EF, which was determined with Simpson's rule: EF = (LVEDV-LVESV)/LVEDV, where: LVEDV - left-ventricular end-diastolic volume, and LVESV - left-ventricular end-systolic volume [8]. Left ventricular end-diastolic diameter (LVEDD) and left ventricular end-systolic diameter (LVESD) were measured from M-mode tracings. Parameters of flows were assessed in Doppler examination (continuous, pulsed and colour) [8].

Diastolic function of the left ventricle was assessed using the parameters of mitral inflow registered with pulsed wave (PW) Doppler in 4-cavity apical projection and diastolic speed values of movement of the mitral ring registered with tissue Doppler imaging.

### Statistical analysis

The STATISTICA software (StatSoft, Warsaw, Poland) package was used for statistical analysis. All values presented are the mean ± standard deviation for continuous variables and the percentage of total patients for categorical variables. The Shapiro-Wilk test was used to assess normality of distribution of the analyzed features. To compare groups Student's t test or two-way analysis of variance (ANOVA) for continuous and discrete variables with normal distribution and non-parametric Mann-Whitney U test if the distribution was not normal were applied. For categorical variables chi-square test or Fisher's test for small samples were applied for comparisons. For quantitative variables (continuous and discrete) to check correlations between variables Spearman's rank correlation coefficient was used. Results were considered statistically significant at p < 0.05.

## Results

### Characteristics of patients with and without AF

Baseline patient demographics and characteristics are presented in Table 1. There were no significant changes between groups (AF vs. non-AF) according to HF signs after eligibility. As the most frequent finding, 43% of patients from non-AF group and 52% from the AF group suffered from dyspnoea. In patients with co-existing AF, warfarin and digoxin treatment were applied significantly more frequently (96% vs 6.8%, p = 0.03 and 56% vs. 9%, p = 0.03, respectively). During the study, one patient needed reduction of carvedilol doses, in two patients digoxin was administered after 8 weeks, and angiotensin converting enzyme inhibitor (ACE-I) was weaned because of hyponatremia in one patient. No patient had left ventricular hypertrophy, defined as enlargement of the diastolic dimension of the interventricular septum and/or posterior wall>1.2 cm.

**Table 1:** Characteristics of the groups.

	ALL PATIENTS		
		
PARAMETER	Group with AF n = 25	Group without AF n = 44	*p*
Age	57 ± 10.26	59 ± 13	ns

-female -male	3 22	7 37	***0.04***

diabetes mellitus	7 (28%)	9 (20%)	ns

cigarette smoking	4 (16%)	4 (9%)	ns

BMI	28 ± 4	25 ± 3	ns

NYHA class median	2	3	ns

**Heart failure assessment**			

dyspnoea (1)*	13 (52%)	18 (43%)	ns

oedema (1)	6 (25%)	4 (9%)	ns

pulmonary hemostasis1	5 (21%)	3 (7%)	ns

6-MWT (1)	405 ± 71	381.9 ± 96	ns

6-MWT (2)*	434 ± 109	419.1 ± 114	ns

HR mean	78	77	ns

**Pharmacological treatment**			

atorvastatin	17 (68%)	27 (61%)	ns

carvedilol	23 (92%)	38 (86%)	ns

ACE-I	23 (92%)	42 (95%)	ns

ARB	2 (8%)	3 (7%)	ns

spironolactone/eplerenone	22 (88%)	40 (93%)	ns

diuretics	22 (88%)	42 (95%)	ns

aspirin	9 (36%)	20 (45%)	ns

digoxin	14 (56%)	4 (9%)	***0.001***

warfarin	24 (96%)	3 (6.8%)	***0.001***

insulin	1 (4%)	3 (7%)	ns

oral hypoglycaemics	3 (12%)	4 (9%)	ns

**Echocardiographic characteristics**			

LA enlargement [cm]	22 (88%)	37 (84%)	ns

LVEDD [cm]	7.3 ± 0.9	7.0 ± 0.8	ns

LVESD [cm]	6 ± 2	5.7 ± 1	ns

diastolic dysfunction	2 (8%)	6 (13.6%)	ns

LVEDV[cm]	240.24 ± 99	200.55 ± 57	ns

LVESV [cm]	175.11 ± 86	147.33 ± 56.58	ns

EF (%)	30%	29%	ns

mitral incompetence I II III	11 (44%) 5 (20%) 3 (12%)	15 (34%) 16 (36%) 10 (24%)	ns

pulmonary hypertension	7 (28%)	13 (30%)	ns

**Biochemical parameters**			

TNF-α (1) [pg/ml]	20.2 ± 28	16.5 ± 17	ns

IL-6 (1) [pg/ml]	20.6 ± 20	13.8 ± 10	ns

IL-10 (1) [pg/ml]	14.2 ± 12	24.9 ± 41	ns

NT-proBNP (1) [pg/ml]	2669.4 ± 2192	1540.7 ± 1513	***0.02***

* (1) variables assessed after inclusion, (2) variables assessed after 2 months

ABBREVIATIONS: BMI - body mass index; HR - heart rate; AF - atrial fibrillation; NYHA - New York Heart Association; 6-MWT - 6-minute walk test, ACE-I - angiotensin-converting enzyme inhibitor; ARB - angiotensin II receptor antagonist; LA - left atrium; LVEDD - left ventricular end-diastolic diameter; LVEF - left ventricular ejection fraction; LVESD - left ventricular end-systolic diameter; LVEDV - left ventricular end-diastolic volume; LVESV - left ventricular end-systolic volume, TNF-a - tumour necrosis factor-alpha; IL - interleukin; NT-proBNP - N-terminal pro-brain natriuretic peptide.

Dimension of left atrium was assessed in longitudinal parasternal projection (M-mode) (>4.0 cm LA enlargement was recognized). No patients had diagnosed restrictive mitral inflow pattern. No significant differences concerning LVEF, left chamber size and volume, or the degree of mitral incompetence were observed between the examined groups. In the whole population of patients, the NT-proBNP level was significantly higher in the group with AF (2669.4 ± 2192 vs 1540.7 ± 1513; p = 0.02).

### Comparison of patients with DCM treated with atorvastatin with and without AF

**Figure 1:**
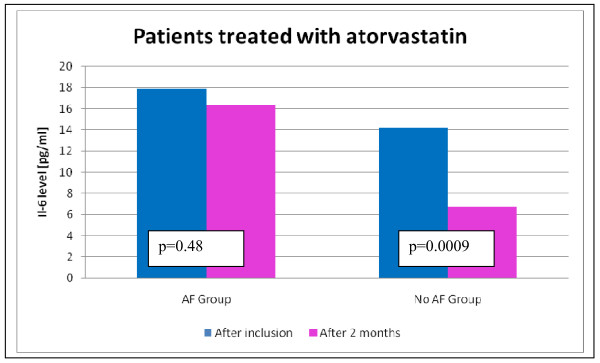
**The comparison of IL-6 level in AF and non-AF patients treated with atorvastatin**.

We divided patients who were treated with atorvastatin according to co-existence or not of AF and compared these groups. We assessed inflammatory state, HF signs, NT-proBNP level and echocardiographic parameters before and after 2 months of statin therapy, which is presented in Table 2. In non-AF patients, a higher reduction of IL-6 concentration (from 16.32 ± 12.76 to 6.74 ± 5.45; p < 0.001) as a result of statin treatment was observed than in patients with AF (17.91 ± 17.42 to 14.16 ± 13.40; p = 0.48) (Figure 1). In the group with AF as well as in non-AF group, a decrease in NT-proBNP concentration was observed. In patients with DCM and co-existing AF statin treatment, higher values of NT-proBNP and IL-6 were observed than in patients without AF (1530 ± 1054 vs 1006 ± 1195, p = 0.04 and 14.16 ± 13.40 vs 6.74 ± 5.45, p = 0.02, respectively). There were no statistically significant differences according to HF symptoms, echocardiographic parameters, 6-MWT and NYHA class between analysed groups.

**Table 2:** Comparison of patients with DCM treated with atorvastatin with and without AF.

	Patients treated with ATORVASTATIN		
		
PARAMETER	AF Group n = 17	No AF Group n = 27	*p*
***Signs of heart failure***			

dyspnoea (1)*	9 (53%)	8 (30%)	ns

dyspnoea (2)*	5 (29%)	7 (26%)	ns

oedema (1)	4 (23.5)%	2 (7.5%)	ns

oedema (2)	2 (11.7%)	1 (4%)	ns

pulmonary haemostasis (1)	2 (11.7%)	2 (7.5%)	ns

pulmonary haemostasis (2)	1 (5.8%)	1 (4%)	ns

***Markers of inflammation***	mean ± standard deviation	mean ± standard deviation	

TNF-α (1) [pg/ml]	21.54 ± 31.33	20.30 ± 21	0.25

TNF-α (2) [pg/ml]	15.21 ± 20.15	9.56 ± 10.72	0.25

IL-6 (1) [pg/ml]	17.91 ± 17.42	16.32 ± 12.76	0.72

IL-6 (2) [pg/ml]	14.16 ± 13.40	6.74 ± 5.45	***0.02***

IL-10 (1) [pg/ml]	9.98 ± 9.27	19.51 ± 32	0.28

IL-10 (2) [pg/ml]	13.21 ± 9.53	31.40 ± 68.95	0.74

***Heart Failure Assessment***			

NT-proBNP (1) [pg/ml]	2002.93 ± 1391.52	1518.87 ± 1732.83	0.14

NT-proBNP (2) [pg/ml]	1530.62 ± 1054.27	1006.76 ± 1195.13	***0.04***

NYHA class (1) median	2	3	0.35

NYHA class (2) median	2	2	0.87

EF (1) [%]	31.13 ± 7	29.82 ± 7.90	0.62

EF (2) [%]	32 ± 8	32.00 ± 8.44	0.84

6-MWT (1)	419.23 ± 72	376.09 ± 108.54	0.14

6-MWT (2)	466.25 ± 104	441.84 ± 118	0.67

* (1) variables assessed after inclusion, (2) variables assessed after 2 months.

ABBREVIATIONS: TNF-α - tumour necrosis factor-alpha; IL - interleukin, 6-MWT - 6-minute walk test; EF - ejection fraction; NYHA - New York Heart Association; NT-proBNP - N-terminal pro-brain natriuretic peptide.

### Factors which influenced NT-proBNP level in all patients - assessment after inclusion and after 2 months of therapy

Two months after inclusion in the study, a statistically significant influence on NT-proBNP level was shown by: atorvastatin treatment (connected with lower level of NT-proBNP; p = 0.01), renal insufficiency and necessity of digoxin treatment (connected with higher levels of NT-proBNP). AF was associated with higher level of NT-proBNP at the beginning of observation but this influence was not observed after two months of atorvastatin treatment (table 3).

**Table 3:** Factors (non-measurable variables) which significantly influence NT-proBNP level.

Factors which significantly affect NT-proBNP	after inclusion	after 2 months
atorvastatin treatment	-	*p = 0.01*

renal insufficiency	-	*p = 0.04*

atrial fibrillation	*p = 0.03*	-

digoxin therapy	*p = 0.001*	*p = 0.001*

ABBREVIATIONS: NT-proBNP - N-terminal pro-brain natriuretic peptide.

## Discussion

AF occurs in congestive HF with the frequency of 15 to 30% [12]. In the study of Brembilla-Perrot et al. [19], the occurrence of fixed AF was not a factor inducing the occurrence of fixed ventricular tachycardia, nor did it influence the prognosis in patients with DCM. In the research by Parthenakis et al. [20], the occurrence of AF in patients with DCM was related to higher concentration of IL-6 (p = 0.001), TNF-α (p = 0.002), soluble tumor necrosis factor receptor inhibitor (sTNFRI) (p = 0.023), N-terminal atrial natriuretic factor (NT-ANP) (p < 0.001), NT-proBNP (p = 0.003), decreased exercise tolerance and lower maximum oxygen consumption at exercise peak compared to patients in sinus rhythm. Wojtkowska et al. [21], in patients with AF co-occurring with HF, showed lower physical efficiency as well as a higher number of hospitalizations. However, AF was not an independent risk factor of death. In the available literature, it was determined that after slower heart rhythm is obtained in tachyarrhythmic cardiomyopathy, a considerable improvement in left ventricular function is also obtained contrary to other kinds of cardiomyopathy in which the prognosis is bad [22]. In order not to falsify the results of assessment of atorvastatin influence, the research included the patients (in the research group as well as in the control group) who were treated optimally pharmacologically for 3 weeks before the qualification. The average rhythm rate did not differ after inclusion in the research between the statin group and the group without statin and it was 78 beats/minute. The research did not include patients with tachyarrhythmia. After two months from inclusion in the research, no significant differences were found in heart rate within the groups and between the groups; pharmacological treatment was not modified during that time either. In order to assess whether the occurrence of AF influences the effectiveness of statin treatment and prognoses for patients with DCM, and if it does, how it influences them, patients with AF and non-AF patients were compared within the whole population and within the statin group. In patients with DCM and AF, higher levels of NT-proBNP were only determined when they were included in the research [22]. The influence of AF on the level of NT-proBNP was also confirmed by data from the literature [20,23]. In the author's own research, as a result of statin treatment, patients with AF obtained a considerably lower reduction of NT-proBNP and IL-6 levels than non-AF patients. No differences in echocardiographic parameters and clinical assessment were determined (NYHA class, 6-MWT, HF symptoms) in the groups with AF and without AF after statin treatment. The results of the MADIT-II (Multicenter Automated Defibrillator Implantation Trial II) [24] and DEFINITE (Defibrillators in Non-ischaemic Cardiomyopathy Treatment Evaluation) [25] studies showed a considerable reduction of morbidity rate and ventricular arrhythmia in patients taking statins. In the ADVANCENT^SM ^(National Registry to Advance Heart Health) registry, treatment with statins was connected with a considerable reduction of AF occurrence [26]. The registry confirmed that anti-arrhythmic influence of statins may be a result of their anti-ischaemic properties, stabilization of cell membranes, improvement of autonomous nervous system function, inhibition of triphosphatase Rac1 guanosine, counteracting the remodelling of the left ventricle and anti-inflammatory properties of statins [26]. Based on the results of the author's research, it may be stated that advantageous effects were to a small degree a consequence of statins' immunomodulating activity - lower reduction of inflammatory cytokine levels in the group with AF. Factors influencing levels of NT-proBNP were assessed as well. The only cytokine among those examined the concentration of which positively correlates with the levels of NT-proBNP turned out to be IL-6. We also showed that cytokine activity was higher in the group of patients with co-occurring AF. As a result of statin treatment, a considerably lower reduction of IL-6 levels was obtained in patients with AF than in the non-AF group. In the research by Streitner et al., higher levels of IL-6 were related to occurrence of malignant ventricular arrhythmia in patients with an ICD [27]. The importance of IL-6 was also confirmed in the study of Haugen et al., where in the group of elderly patients increased levels of inflammatory cytokines were determined; however, only the concentration of IL-6 was a predictor of early death [28].

Our study has several limitations. The most important is the small number of patients in both groups (AF and non-AF), and the fact it is not a placebo-controlled study.

In conclusion, in patients with DCM and co-existing AF, a weaker effect of atorvastatin concerning the reduction of IL-6 and NT-proBNP concentration was observed than in patients without AF. However, this interesting data on the decreased pleiotropic activity of atorvastatin in patients with DCM and AF, has to be confirmed in a placebo-controlled study with a larger number of patients [29-31].

## Competing interests

The authors declare that they have no competing interests.

## Authors' contributions

ABD planned the study protocol, took care about the patients and coordinate the research, JHG was a medical patients consultant and participated in the sequence alignment, JR collect data and drafted the manuscript, MM carried out echocardiographic assessment, RD participated in the design of the study and performed the statistical analysis, WA participated in the design of the study and revised it critically for important intellectual content and MB conceived of the study, and participated in its design, and coordination. All authors read and approved the final manuscript.

## References

[B1] Viswanathan MN Page RL Pharmacological therapy for atrial fibrillation: current options and new agents Expert Opin Investig Drugs 2009 18 4 417 431 10.1517/13543780902773410 19278302

[B2] Aronow WS Banach M Atrial Fibrillation: The New Epidemic of the Ageing World J Atrial Fibrillation 2009 1 337 361 10.4022/jafib.154 PMC5398780 28496617

[B3] Grönefeld GC Hohnloser SH Heart failure complicated by atrial fibrillation: mechanistic, prognostic, and therapeutic implications J Cardiovasc Pharmacol Ther 2003 8 2 107 113 10.1177/107424840300800203 12808483

[B4] Banach M Kourliouros A Reinhart KM Benussi S Mikhailidis DP Jahangiri M Baker W Galanti A Rysz J Camm JA White CM Alfieri O Postoperative atrial fibrillation - what do we really know? Curr Vasc Pharmacol 2010 in press 19538179 10.2174/157016110791330807

[B5] Kaski JP Elliott P ESC Working Group The classification concept of the ESC Working Group on myocardial and pericardial diseases for dilated cardiomyopathy Herz 2007 32 6 446 51 10.1007/s00059-007-3045-5 17882369

[B6] Kellwellis-Opara A Dörner A Poller WC Noutsias M Kühl U Schultheiss HP Pauschinger M Autoimmunological features in inflammatory cardiomyopathy Clin Res Cardiol 2007 96 469 480 10.1007/s00392-007-0524-x 17503113

[B7] Seiler J Stevenson WG Atrial fibrillation in congestive heart failure Cardiol Rev 2010 18 1 38 50 10.1097/CRD.0b013e3181c21cff 20010337

[B8] Bielecka-Dabrowa A Goch JH Mikhailidis DP Rysz J Maciejewski M Banach M The influence of atorvastatin on parameters of inflammation and function of the left ventricle in patients with dilated cardiomyopathy Med Sci Monit 2009 15 12 MS12 23 19946241

[B9] Banach M Mikhailidis DP Kjeldsen SE Rysz J Time for new indications for statins? Med Sci Monit 2009 15 12 MS1 5 19946240

[B10] Wassmann S Laufs U Baumer AT Müller K Konkol Ch Sauer H Böhm M Nickenig G Inhibition of geranylgeranylation reduces angiotensin II-mediated free radical production in vascular smooth muscle cells: involvement of angiotensin AT1 receptor expression and Rac1 GTPase Mol Pharmacol 2001 59 646 654 11179461 10.1124/mol.59.3.646

[B11] Brown JH Del Re DP Sussman MA The Rac and Rho hall of fame: a decade of hypertrophic signaling hits Circ Res 2006 98 730 742 10.1161/01.RES.0000216039.75913.9e 16574914

[B12] Wainwright G Mascitelli L Goldstein MR Cholesterol-lowering therapy and cell membranes. Stable plaque at the expense of unstable membranes? Arch Med Sci 2009 5 3 289 295

[B13] Liao JK Isoprenoids as mediators of the biological effects of statins J Clin Invest 2002 110 285 288 12163444 10.1172/JCI16421 PMC151100

[B14] Hernandez-Perera O Perez-Sala D Navarro-Antolin J Sánchez-Pascuala R Hernández G Díaz C Lamas S Effects of the 3-hydroxy-3-methylglutaryl-CoA reductase inhibitors, atorvastatin and simvastatin, on the expression of endothelin-1 and endothelial nitric oxide synthase in vascular endothelial cells J Clin Invest 1998 101 2711 2719 10.1172/JCI1500 9637705 PMC508862

[B15] Kowalski J Barylski M Banach M Grycewicz J Irzmański R Pawlicki L Neutrophil superoxide anion generation during atorvastatin and fluvastatin therapy used in coronary heart disease primary prevention J Cardiovasc Pharmacol 2006 48 4 143 7 10.1097/01.fjc.0000246150.52382.07 17086091

[B16] Laufs U La Fata V Plutzky J Upregulation of endothelial nitric oxide synthase by HMG CoA reductase inhibitors Circulation 1998 97 1129 1135 9537338 10.1161/01.cir.97.12.1129

[B17] Tousoulis D Antoniades C Bosinakou E Kotsopoulou M Tsioufis C Tentolouris C Trikas A Pitsavos C Stefanadis C Effects of atorvastatin on reactive hyperaemia and the thrombosis-fibrinolysis system in patients with heart failure Heart 2005 91 27 31 10.1136/hrt.2003.027110 15604328 PMC1768647

[B18] Engvall E Perlman P Enzyme-linked immunosorbent assay (ELISA). Quantative assay of immunoglobulin G Immunochemistry 1971 9 871 874 10.1016/0019-2791(71)90454-X 5135623

[B19] Brembilla-Perrot B Marçon O Chometon F Groben L Claudon O Terrier de la Chaise A Louis P Blangy H Sadoul N Selton O Ammar S Abbas M Juillière Y Significance of permanent atrial fibrillation in idiopathic dilated cardiomyopathy Ann Cardiol Angeiol (Paris) 2007 56 3 107 110 10.1016/j.ancard.2007.02.003 17572169

[B20] Parthenakis FI Patrianakos AP Skalidis EI Diakakis GF Zacharis EA Chlouverakis G Karalis IK Vardas PE Atrial fibrillation is associated with increased neurohumoral activation and reduced exercise tolerance in patients with non-ischemic dilated cardiomyopathy Int J Cardiol 2007 118 2 206 214 10.1016/j.ijcard.2006.03.090 17027102

[B21] Wojtkowska I Sobkowicz B Musiał WJ Kozuch M Persistent atrial fibrillation as a prognostic factor of outcome in patients with advanced heart failure Kardiol Pol 2006 64 8 777 783 16981052

[B22] Calò L De Ruvo E Sette A Sciarra L Scioli R Sebastiani F Topai M Iulianella R Navone G Lioy E Gaita F Tachycardia-induced cardiomyopathy: mechanisms of heart failure and clinical implications J Cardiovasc Med (Hagerstown) 2007 8 3 138 143 17312430 10.2459/01.JCM.0000260841.30415.62

[B23] Piechota M Banach M Jacoń A Rysz J Natriuretic peptides in cardiovascular diseases Cell Mol Biol Lett 2008 13 2 155 81 10.2478/s11658-007-0046-6 17965966 PMC6275881

[B24] Baigent C Keech A Kearney PM Blackwell L Buck G Pollicino C Kirby A Sourjina T Peto R Collins R Simes R Cholesterol Treatment Trialists' (CTT) Collaborators. Efficacy and safety of cholesterol-lowering treatment: prospective meta-analysis of data from 90,056 participants in 14 randomised trials of statins Lancet 2005 366 1267 1278 10.1016/S0140-6736(05)67394-1 16214597

[B25] Goldberger JJ Subacius H Schaechter A Howard A Berger R Shalaby A Levine J Kadish AH DEFINITE Investigators Effects of statin therapy on arrhythmic events and survival in patients with nonischemic dilated cardiomyopathy J Am Coll Cardiol 2006 48 1228 1233 10.1016/j.jacc.2006.05.053 16979011

[B26] Hanna IR Heeke B Bush H Brosius L King-Hageman D Dudley SC Jr Beshai JF Langberg JJ Lipid-lowering drug use is associated with reduced prevalence of atrial fibrillation in patients with left ventricular systolic dysfunction Heart Rhythm 2006 3 881 886 10.1016/j.hrthm.2006.05.010 16876733 PMC3164215

[B27] Streitner F Kuschyk J Veltmann C Brueckmann M Streitner I Brade J Neumaier M Bertsch T Schumacher B Borggrefe M Wolpert C Prospective study of interleukin-6 and the risk of malignant ventricular tachyarrhythmia in ICD-recipients--a pilot study Cytokine 2007 40 1 30 34 10.1016/j.cyto.2007.07.187 17851087

[B28] Haugen E Gan LM Isic A Skommevik T Fu M Increased interleukin-6 but not tumour necrosis factor-alpha predicts mortality in the population of elderly heart failure patients Exp Clin Cardiol 2008 13 1 19 24 18650968 PMC2435396

[B29] Banach M Goch JH Ugurlucan M Mariscalco G Rysz J Statins in the prevention of postoperative atrial fibrillation: is there really no effect? Am Heart J 2008 155 6 e53 10.1016/j.ahj.2008.03.008 18513502

[B30] Guo HM Anti-arrhythmic effects of statins: a hypothesis remains to be tested Heart Rhythm 2008 5 4 511 2 10.1016/j.hrthm.2008.01.033 18362017

[B31] Zimmermann AV Doehner W Pérez AV Schmidt H Volk HD Anker SD Rauchhaus M The relationship between high-density lipoprotein, bacterial lipopolysaccharide, and tumour necrosis factor-α in patients with acute decompensated heart failure Arch Med Sci 2008 4 4 380 385

